# Is the Performance of a Specialist Herbivore Affected by Female Choices and the Adaptability of the Offspring?

**DOI:** 10.1371/journal.pone.0143389

**Published:** 2015-11-23

**Authors:** Tarcísio Visintin da Silva Galdino, Marcelo Coutinho Picanço, Dalton Oliveira Ferreira, Geverson Aelton Resende Silva, Thadeu Carlos de Souza, Gerson Adriano Silva

**Affiliations:** 1 Department of Plant Science, Federal University of Viçosa, Viçosa, MG, Brazil; 2 Department of Entomology, Federal University of Viçosa, Viçosa, MG, Brazil; Pennsylvania State University, UNITED STATES

## Abstract

The performance of herbivorous insects is related to the locations of defenses and nutrients found in the different plant organs on which they feed. In this context, the females of herbivorous insect species select certain parts of the plant where their offspring can develop well. In addition, their offspring can adapt to plant defenses. A system where these ecological relationships can be studied occurs in the specialist herbivore, *Tuta absoluta*, on tomato plants. In our experiments we evaluated: (i) the performance of the herbivore *T*. *absoluta* in relation to the tomato plant parts on which their offspring had fed, (ii) the spatial distribution of the insect stages on the plant canopy and (iii) the larval resistance to starvation and their walking speed at different instar stages. We found that the *T*. *absoluta* females preferred to lay their eggs in the tomato plant parts where their offspring had greater chances of success. We verified that the *T*. *absoluta* females laid their eggs on both sides of the leaves to better exploit resources. We also observed that the older larvae (3^rd^ and 4^th^ instars) moved to the most nutritious parts of the plant, thus increasing their performance. The *T*. *absoluta* females and offspring (larvae) were capable of identifying plant sites where their chances of better performance were higher. Additionally, their offspring (larvae) spread across the plant to better exploit the available plant nutrients. These behavioral strategies of *T*. *absoluta* facilitate improvement in their performance after acquiring better resources, which help reduce their mortality by preventing the stimulation of plant defense compounds and the action of natural enemies.

## Introduction

Insects are the most diverse group of living organisms on the planet [1]. To succeed, insects have adopted strategies to adapt to different environments. One of the most economically important groups of insects includes herbivorous species, whose success has resulted in several great losses to crops. Herbivorous insects exhibit complex strategies to adapt to prevalent adverse environmental conditions, including the activities of their natural enemies, climatic hazards and host plant defenses [2–5]. In this context, the spatial distribution of these insects in the plant canopy and their performance are dependent upon the location of plant defenses in the canopy [3], [6].

Many species of herbivorous insects specialize in feeding upon specific species or particular plant parts [7–9]. In this context, studies on the performance and spatial distribution of insects in the plant canopy can help plan and develop pest control programs on a sustainable basis [9], [10].

During their life cycle, holometabolous herbivorous insects develop via the egg, larva, pupa and adult stages. These insect females are usually winged and select sites that are most favorable for their offspring to survive during the beginning of their life cycle, when the eggs hatch and the larvae feed on the host plant. This is known as the “mothers know best” strategy [11]. In some of insect species, the female chooses a place to oviposit in a way that their offspring will have the best possible development [3] However, not all insect species exploit this strategy, and females can oviposit in places where the food is not suitable for the offspring. In both cases, the larval stage of these herbivorous insects must adapt to overcome the plant defenses and select plant sites with higher nutrient levels. This behavior enables the herbivorous insect to accumulate the energy required to complete its life cycle [3], [4].

Tomato plants (*Solanum lycopersicum*) possess morphological structures and phytochemicals that act as natural defenses against herbivorous insects. In tomatoes, the plant defenses consist of trichomes, toxins, anti-nutritive defensive proteins, protease inhibitors and cellular structures [12], [13]. The quantities in which these defenses are present vary among the different plant parts in tomatoes [13–15].

*Tuta absoluta* (Meyrick) (Lepidoptera: Gelechiidae) is a specialist herbivorous insect that uses the tomato plant as its chief host. This insect is a major pest of tomatoes in South America [16–18]. It was introduced to Europe via Spain and has now dispersed throughout the entirety of this continent. Recently, it was introduced to Africa and Asia, where it has caused great crop losses [19], [20]. The damage caused by this pest is due to its larvae, which mine the leaves and bore through the stems and fruits. This insect is capable of leaving the mine and walking throughout the plant to find other suitable place to feed, which can be one of the plant parts cited above. It can also move in the plant by tunneling through the stems, but this is less common [13], [16–18].

*T*. *absoluta* is a specialist insect, and its development in the tomato plant might be defined by differences in defenses among different parts of the plant canopy. Despite the significance of this subject in the understanding of its ecology, as well as planning control programs for this pest, very little is known about it. Thus, in this study, we aimed to determine whether the biological success of the specialist herbivore *T*. *absoluta* was affected by the selection capacity of the females and the adaptability of their offspring. Therefore, we conducted studies on (i) the performance of the insect in the different plant parts, (ii) its spatial distribution in the plant canopy and (iii) the larval resistance to starvation and their walking speed at different instar stages.

## Material and Methods

This research was conducted at the Federal University of Viçosa, Viçosa, Minas Gerais, Brazil (20°48’45”S, 42°56’15”W, altitude 600 m and tropical climate). This study was divided into three parts.

### Cultivation of the tomato plants

We used plants of the Santa Clara cultivar, which were grown in a greenhouse without insecticides. The cultivation was performed according to Silva and Vale [21], in which the seedlings were grown in a Styrofoam cell tray (68 x 34 x 5 cm) with 200 plugs (each plug with one seedling). The seedlings were then transplanted 24 days after planting to 8-liter plastic pots. The substrate was composed of 2/3 soil (Oxisol) and 1/3 of cattle manure. Fertilizer was added according to agronomical recommendations [21]. Plants were watered three times a day according to the the evapotranspiration of tomato plants in each phase of their development to replace the water consumed by plants and that lost due to evapotranspiration [21]. During the cultivation, the temperature of the greenhouse was kept at approximately 30°C by managing the greenhouse openings.

### *T*. *absoluta* rearing

The insects used in this study were collected from commercial tomato crops in Viçosa. The rearing was performed in the laboratory according to Leite et al. [22] and Galdino et al. [23]. The populations were kept in the laboratory at a temperature of 25 ± 0.5°C, a photoperiod of 12 hours and humidity at 75 ± 1%. Four cages made of wood were used (40 x 40 x 40 cm) and were coated with organza. Each cage contained individuals of similar ages: 1- a cage for adults to lay eggs, 2- a cage with leaves containing eggs and small caterpillars, 3- a cage containing large caterpillars and 4- a cage with pupae and emerging adults. In the adult cage 1, we kept a tomato leaf where the females could lay their eggs. We replaced this leaf daily with a new one, and the leaf with eggs was transferred to the cage 2 containing eggs and small caterpillars. Once the eggs hatched, we offered the larvae new leaves from plants grown in a greenhouse to pupate and become adults. As the caterpillars grew the cage 2 turned into cage 3, and cage 3 in cage 4. The adults from cage 4 were then transferred again to cage 1 to lay eggs.

### Biological performance of *T*. *absoluta* in different plant parts

This trial was conducted to evaluate whether the plant parts themselves could influence the performance of *T*. *absoluta*. We performed this test in the greenhouse, with 20 plants in the reproductive stage. The plants had 22 compound leaves and three clusters with fruit each, and they were 60 days old after transplantation. The experimental design was completely randomized, with 10 repetitions involving two plants each. The first plant was used to evaluate the larval development, while the second plant was used to evaluate the pupation success, oviposition rate of the females and egg hatching. Each organ of the first plant in the apical, middle and basal thirds of the canopy was wrapped in an organza bag (40 x 20 cm). This was done to prevent the *T*. *absoluta* larvae from moving from one organ to another and to enable collection of the pupae.

The treatments included were as follows: 1) apical bud, 2) leaves of the apical third, 3) leaves of the middle third, 4) leaves of the basal third and 5) fruits. Every day the plants were randomly changed in position in the greenhouse.

Each plant organ was then infested with 15 newly hatched larvae. This number was chosen because we noticed that this was the intensity of the pest attack on tomato plants in the field. Daily, the number of living individuals in each larval instar was evaluated.

Pupae from each of the treatments were sexed (see [24]), counted and placed in the second plant of the repetition on the same plant organ where the larvae had developed. Daily, we counted the number of live and dead insects. When the insects reached the adult stage, the number of eggs produced and the rate of egg hatching were counted daily.

From the experimental data, for each repetition we built a Fertility Life Table for *T*. *absoluta* according to Southwood and Henderson [25]. With the data from this Life Table, we calculated the net reproductive rate (R_0_) and generation time (T) [25]. The R_0_ and T data for each plant where the larvae had fed (apical bud, fruits and leaves of the apical, middle and basal thirds) were subjected to the analysis of variance, with p < 0.05 considered significant. Means from the treatments showing significant overall changes were subjected to Tukey's test with significance set at p < 0.05.

### Spatial distribution of the *T*. *absoluta* stages in the plant canopy

Using two greenhouses, we conducted this trial to study the manner in which the *T*. *absoluta* offspring could overcome the defenses of the tomato plants. In the first greenhouse, we cultivated 20 plants with 10 plants per stage. To ensure that we had plants at these different stages, we sowed the seeds with a difference of 35 days between sowing.

The experimental design was completely randomized, with 10 repetitions. Each repetition involved only one plant. The characteristics studied were the plant stages (vegetative and reproductive) and plant organs. In the beginning of the experiment, the plants in the vegetative stage (from the second sowing) had 12 leaves and were 25 days old after transplantation, while the plants in the reproductive stage (from the first sowing) had 22 leaves and three clusters with fruit and were 60 days old after transplantation. In the first greenhouse containing 20 plants (10 in each plant stage), we released 300 adults of unsexed *T*. *absoluta* at 48 hours after their emergence. These plants were maintained for three days in the first greenhouse. The use of insects that were 48 hours of age after their emergence was implemented, as this represented the period of pre-oviposition for *T*. *absoluta* [24]. The rationale for using 15 adults per plant during three days followed from previous study in which we observed that the number of eggs produced was similar to that observed in tomato crops in the field.

After the exposure period of plants to the adult insects, the plants were transferred to the second greenhouse. The second greenhouse was free from *T*. *absoluta* adults so that we could guarantee that the insects on the plants were only from the eggs. Daily, we systematically monitored the number of *T*. *absoluta* stages in the plant canopy. In this step, we counted the number of each insect stage in each plant organ (leaf, leaflet, leaf side, stem, petiole and fruit) in the plant canopy. Additionally, we evaluated the number of insects in the soil and on the stake that supported the plants.

The number of eggs on the compound leaf parts (petiole and each side of the leaflet) and on each leaflet were subjected to analysis of variance (p <0.05) for plants in the vegetative and reproductive stages. The leaf parts were compared by Tukey’s test at p < 0.05 to determine the egg laying preference of *T*. *absoluta* adults for some parts of the leaf. The same procedure was performed on the data analysis of the percentage of pupae in each pupation site (soil, plant or stake) to evaluate the preferred site of pupation.

The egg densities on each leaf position in the canopy were subjected to separate regression analysis for the plants in the vegetative and reproductive stages to determine the preferred position in the plant canopy. The densities of the larvae per plant part as a function of time after commencement of the larval stage were subjected to regression analysis to determine if the larvae stayed at the oviposition site or moved to other parts of the canopy. For plants in the vegetative stage, we fitted curves of the larval densities to the leaves of the apical, middle and basal thirds of the plant canopy. For plants in the reproductive stage, we fitted curves of the number of larvae in the fruits and leaves of three-different thirds of the canopy. We selected the models based on the following criteria: significance of the model (p < 0.05) and the model with the lesser degree of complexity and greater coefficient of determination (R^2^).

To determine if there was a preference by the adults for parts of the plant where the larvae develops better, we performed simple linear regression analysis of the net reproductive rate (these data were derived from a trial on the performance of the insect in different plant parts) versus the number of eggs in the plant parts on which the larvae had fed.

### Ability to survive under starvation and walking speed of the larval instars

We conducted this study to elucidate why different *T*. *absoluta* instars have different behaviors as seen in the previous trials because small larvae did not move to different parts of the canopy, while bigger larvae did. This test was performed in the laboratory under ambient conditions. The treatments were performed on the four larval instars of *T*. *absoluta*. We used newly emerged larvae that had not fed in the instar for this study. We performed two tests, one to evaluate the resistance of the larvae to starvation and the other to evaluate the walking speed of the larvae.

During the trial in which we evaluated the resistance of the larvae to starvation, each repetition included a Petri dish (9 cm diameter x 2 cm height) with ten larvae. We performed 10 repetitions (10 Petri dishes) for each larval instar. No food was provided for the larvae during the test. The numbers of live and dead larvae were then monitored until the last larva died. The results of the ability to survive under starvation (for each larval instar) were subjected to survival analysis using the non-parametric procedure LIFETEST SAS (SAS Institute, Cary, NC, USA) [25][26].

During the course of the trial in which we evaluated the walking speed of the larvae, each repetition consisted of one *T*. *absoluta* larva. Each larva was released at an internode (~10 cm long) of the tomato plant in the reproductive stage. We assessed the distance covered by each larva over a 2 hour period. This evaluation period was used because no deaths occurred during this period of larval starvation. We then calculated the walking speed of each larva and observed whether they fed on the stem.

The mean walking speeds of the larval instars were subjected to analysis of variance (p < 0.05), and the means from the instars showing significant overall changes were subjected to Tukey's test with significance set at p < 0.05. These statistical analyses were performed in SAS Software (SAS Institute, Cary, NC, USA) [26]

## Results

**Fig 1:**
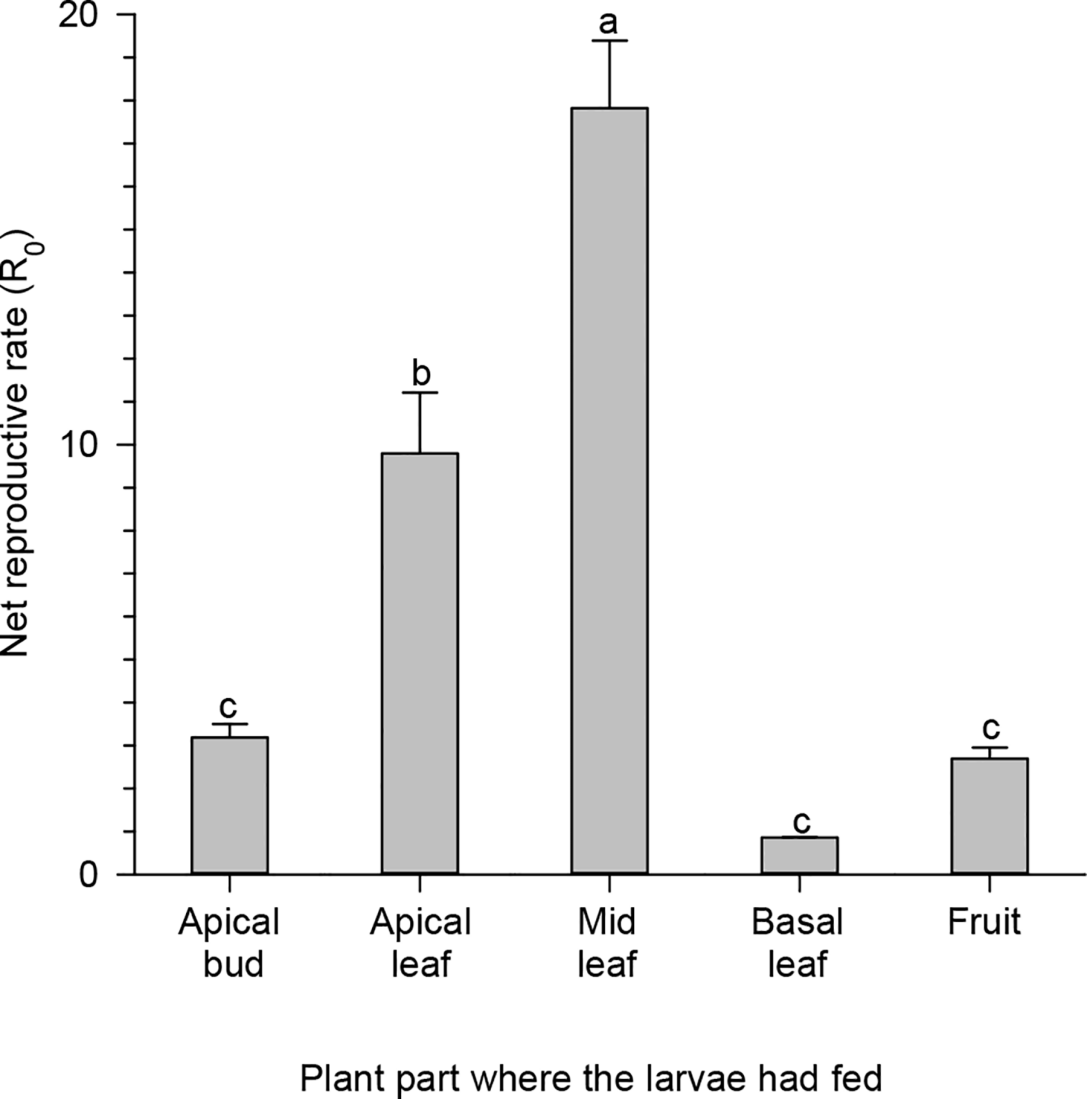
Influence of the plant part on net reproductive rate. The net reproductive rate (R_0_) of *Tuta absoluta* depends on the plant part on which the larvae fed. Histograms followed by the same letter have means that do not differ among themselves by Tukey’s test (p <0.05).

The net reproductive rate (R_0_) of *T*. *absoluta* showed differences depending upon the plant part where the larvae had fed (F_4;45_ = 52.82, p < 0.0001). The highest R_0_ of *T*. *absoluta* was observed to occur when the larvae fed on the leaves of the middle third of the canopy. The lowest R_0_ occurred when the larvae fed on the apical bud, fruits or leaves of the basal third of the canopy. Additionally, the R_0_ of *T*. *absoluta* was between these extremes when the larvae fed on the leaves of the apical third of the canopy (Fig 1). However, we did not verify the effect of the plant part on which the larvae had fed on the generation time of *T*. *absoluta* (F_4;45_ = 0.001, p = 0.9990). Therefore, the success of *T*. *absoluta* was dependent on the plant part where their larvae fed.

**Fig 2:**
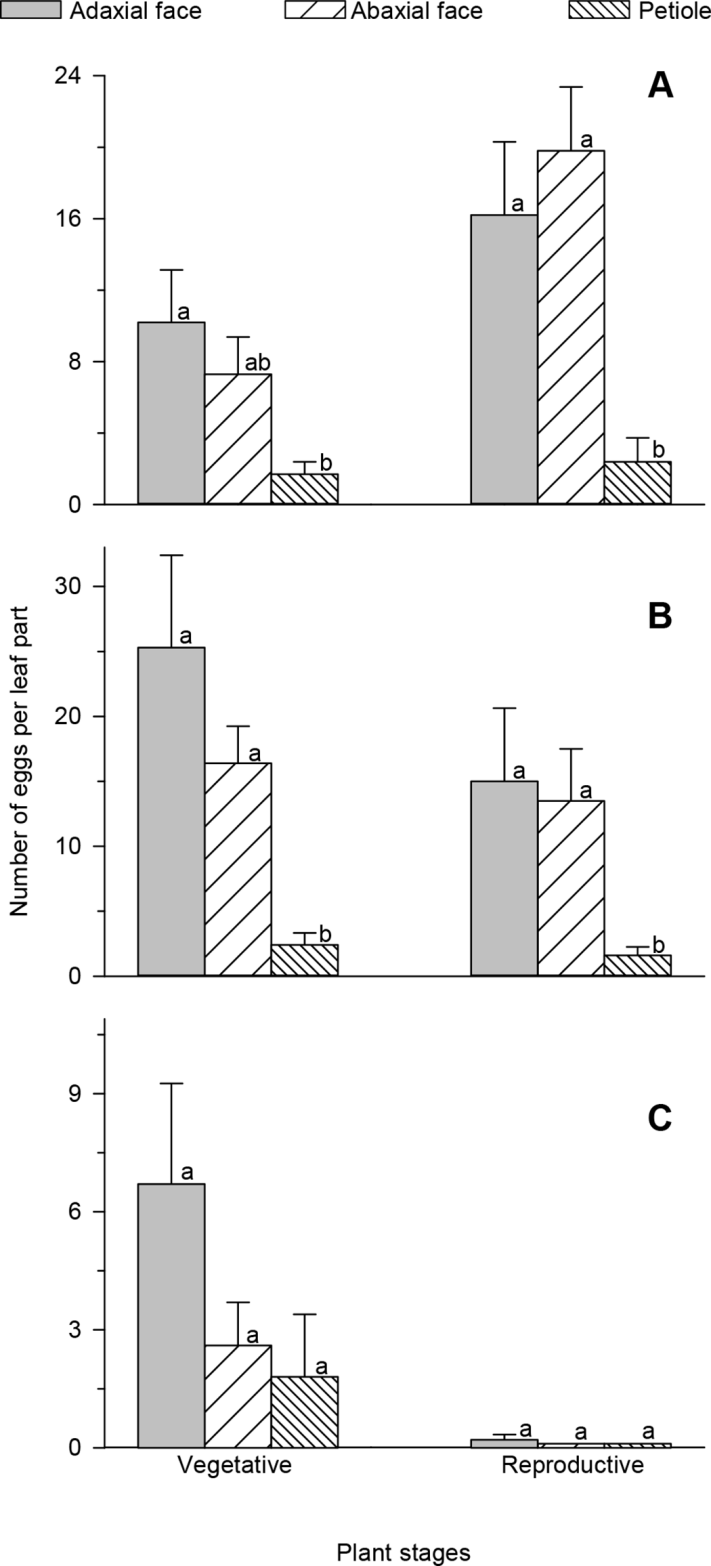
Females preferred leaf sites for oviposition. The number of the eggs (mean ± standard error) of *Tuta absoluta* in the petiole and on the sides of leaves (adaxial and abaxial) in the (A) apical, (B) middle and (C) basal thirds of the plant canopy in the vegetative and reproductive stages. Histograms followed by the same letter have means which do not differ among themselves by Tukey’s test (p <0.05).

Oviposition observed on the adaxial and abaxial surfaces of the leaf blade was higher than that seen on the petioles for the leaves of the apical and middle-thirds of the canopy. In the leaves of these two-thirds, oviposition was similar in the adaxial and abaxial surfaces, a phenomenon that was seen for the plant in both the vegetative and reproductive stages (Fig 2A and 2B). In the leaves of the basal third, the oviposition was similar on both the leaf surfaces and petiole (F_2;27_ = 2.01, p = 0.1529) (Fig 2C). The oviposition of *T*. *absoluta* was similar on the different leaflets of a leaf in the vegetative (F_6;53_ = 1.41, p = 0.2459) and reproductive plant stages (F_6;53_ = 0.96; p = 0.4393).

**Fig 3:**
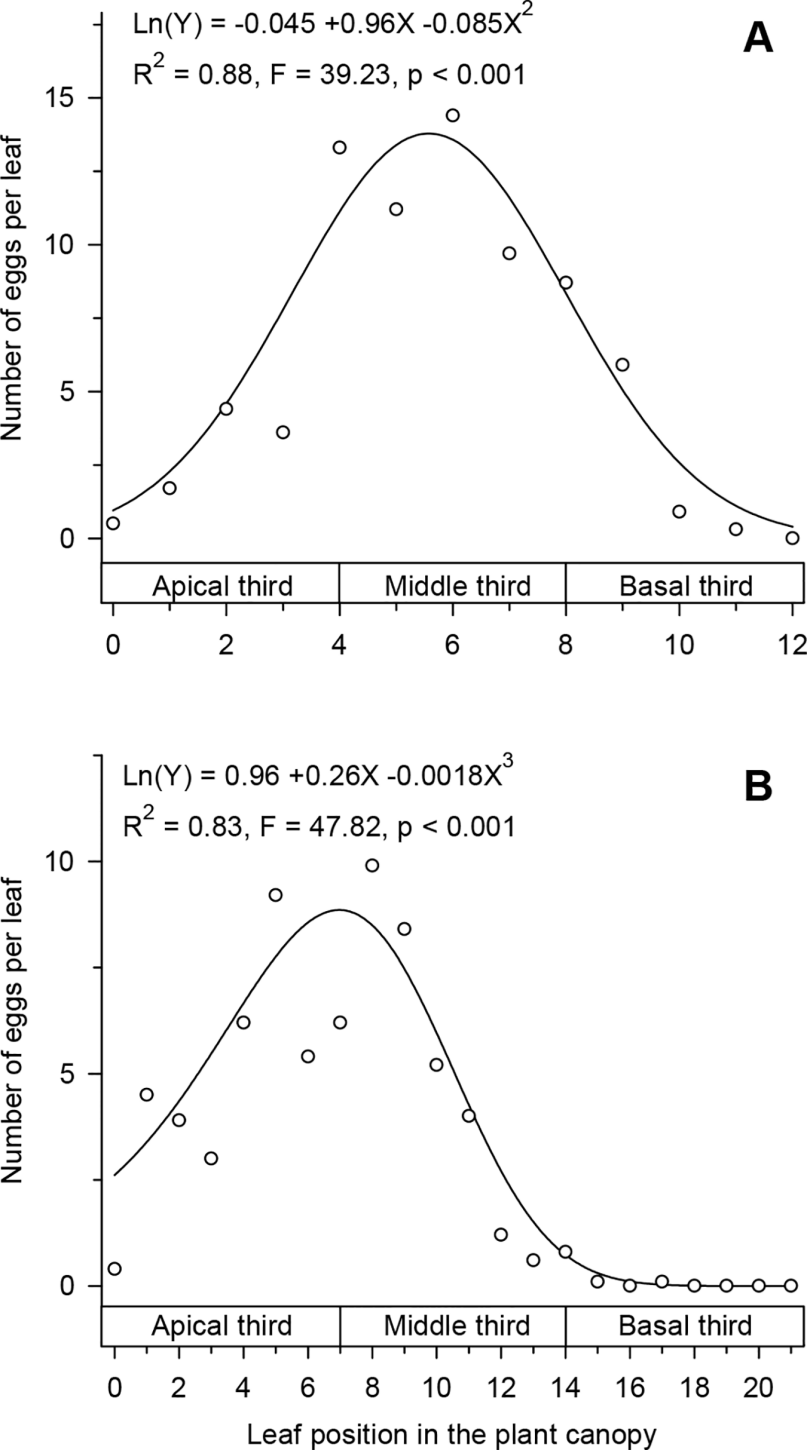
Oviposition of *Tuta absoluta* in relation to the leaf position on the plant canopy. Plants in the (A) vegetative and (B) reproductive stages. On the x-axis: 0 = not expanded leaf at stem apex, 1 = apical-most leaf fully expanded and showing a higher number (12 in Fig 3A and 21 in Fig 3B) = most basal plant leaf.

In the plants at the vegetative stage, the highest oviposition of *T*. *absoluta* was observed to occur on the leaves of the middle third of the canopy (Fig 3A), while in the plants in the reproductive stage, the highest oviposition of *T*. *absoluta* occurred on the leaves between the apical and middle thirds of the canopy (Fig 3B). Thus, the *T*. *absoluta* oviposition basically occurred on fully developed leaves and those that had not become senescent, as most apical leaves were expanding their leaf blades while the basal leaves were becoming senescent.

**Fig 4:**
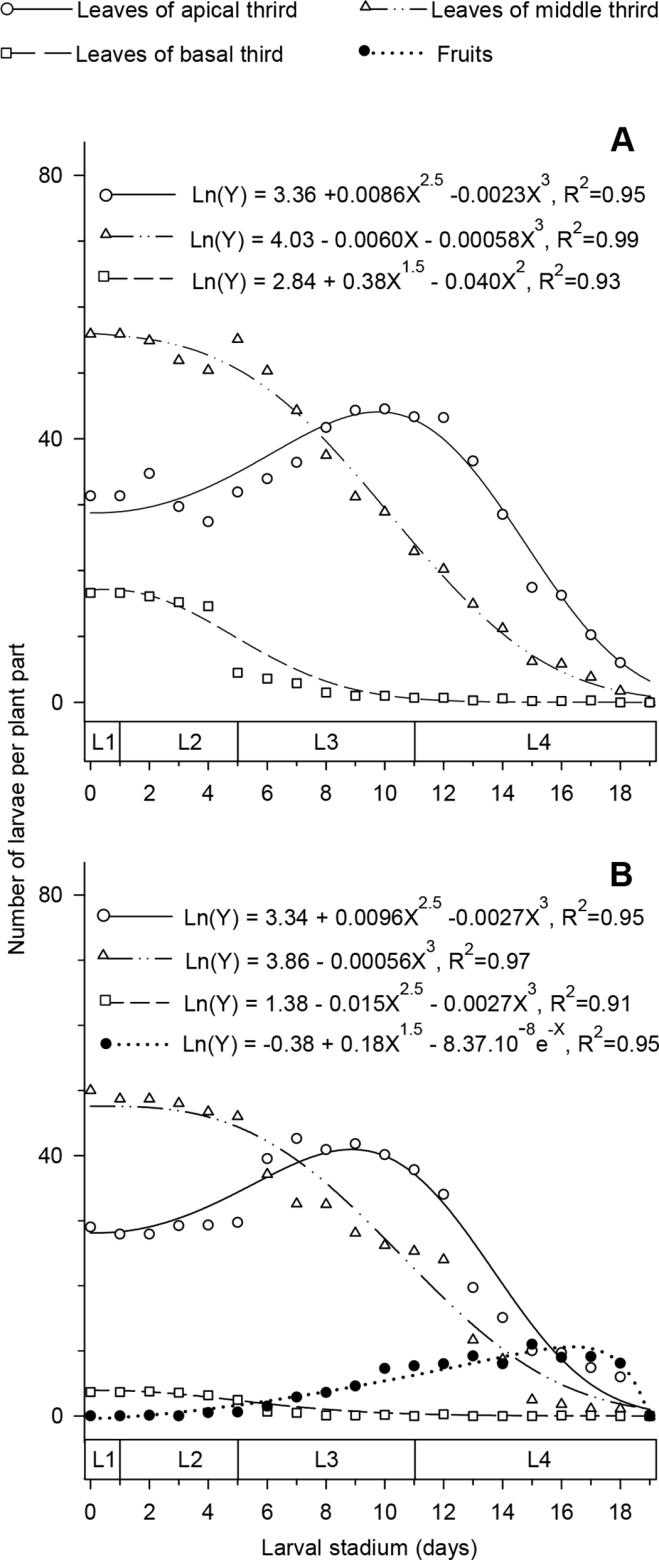
Number of larvae in different thirds of the canopy over time. Number of larvae in the first (L1), second (L2), third (L3) and fourth (L4) instars of *Tuta absoluta* on the leaves in the apical, middle and basal thirds of the canopy and fruit of plants in the (A) vegetative (B) reproductive stages. All fitted models were significant by F test (p <0.05).

At the beginning of the larval stage, the number of larvae was higher in the leaves from the middle third of the canopy. This number was intermediate in the apical third and lower in the basal third of the canopy of plants in both the vegetative and reproductive stages. The number of larvae on the leaves remained constant in the first four days of larval development. Over time, the number of larvae on the leaves of the basal and middle thirds of the canopy decreased, although on the leaves of the apical third of the canopy, the number of larvae increased over time to reach a maximum between the 9^th^ and 10^th^ day after the beginning of larval development. After 8 (plants in vegetative stage) and 7 (plants in the reproductive stage) days from the beginning of larval development, the highest number of larvae was observed on the leaves of the apical third of the canopy (Fig 4A and 4B). We observed the *T*. *absoluta* larvae attacking the fruits only after the 4^th^ day of larval development. At this time, the larvae were in the beginning of the third instar stage. The maximum number of larvae in the fruits occurred on the 16th day of larval development (Fig 4B).

**Fig 5:**
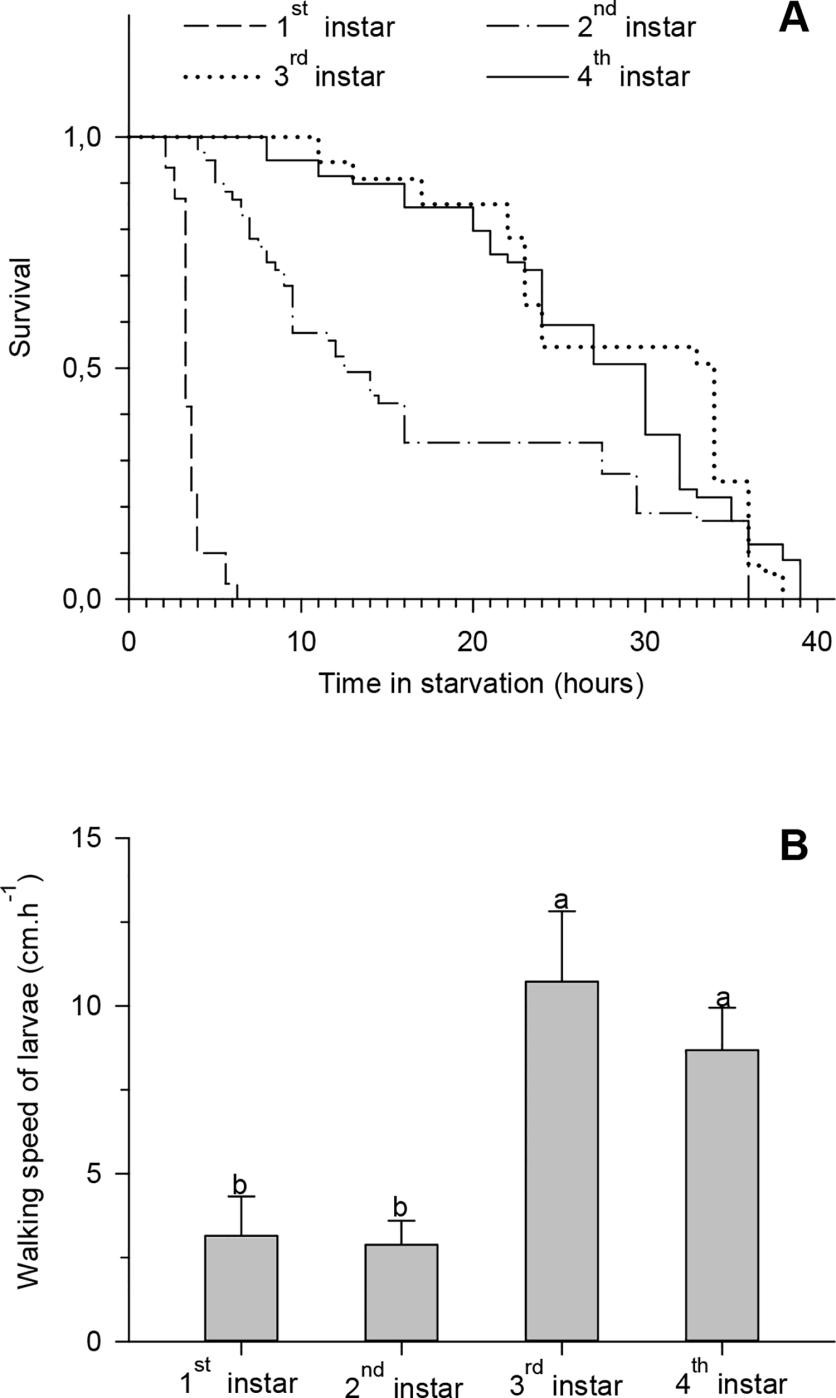
(A) Survival curves under starvation and (B) walking speed of the different larval instars. Fig A represents the survival curves of different *Tuta absoluta* instars with no food supply (non-parametric procedure LIFETEST). In Fig B, histograms followed by the same letter have means that do not differ among themselves by Tukey’s test (p <0.05).

The 3^rd^ and 4^th^ instar larvae were more tolerant to starvation, while the 1st instar larvae were the most susceptible. In contrast, the 2^nd^ instar larvae showed moderate tolerance to starvation (Fig 5A). The larvae of the 3^rd^ and 4^th^ instars showed higher walking speed than the larvae of the 1^st^ and 2^nd^ instars (Fig 5B). The larvae did not feed during the two hour experiment.

**Fig 6:**
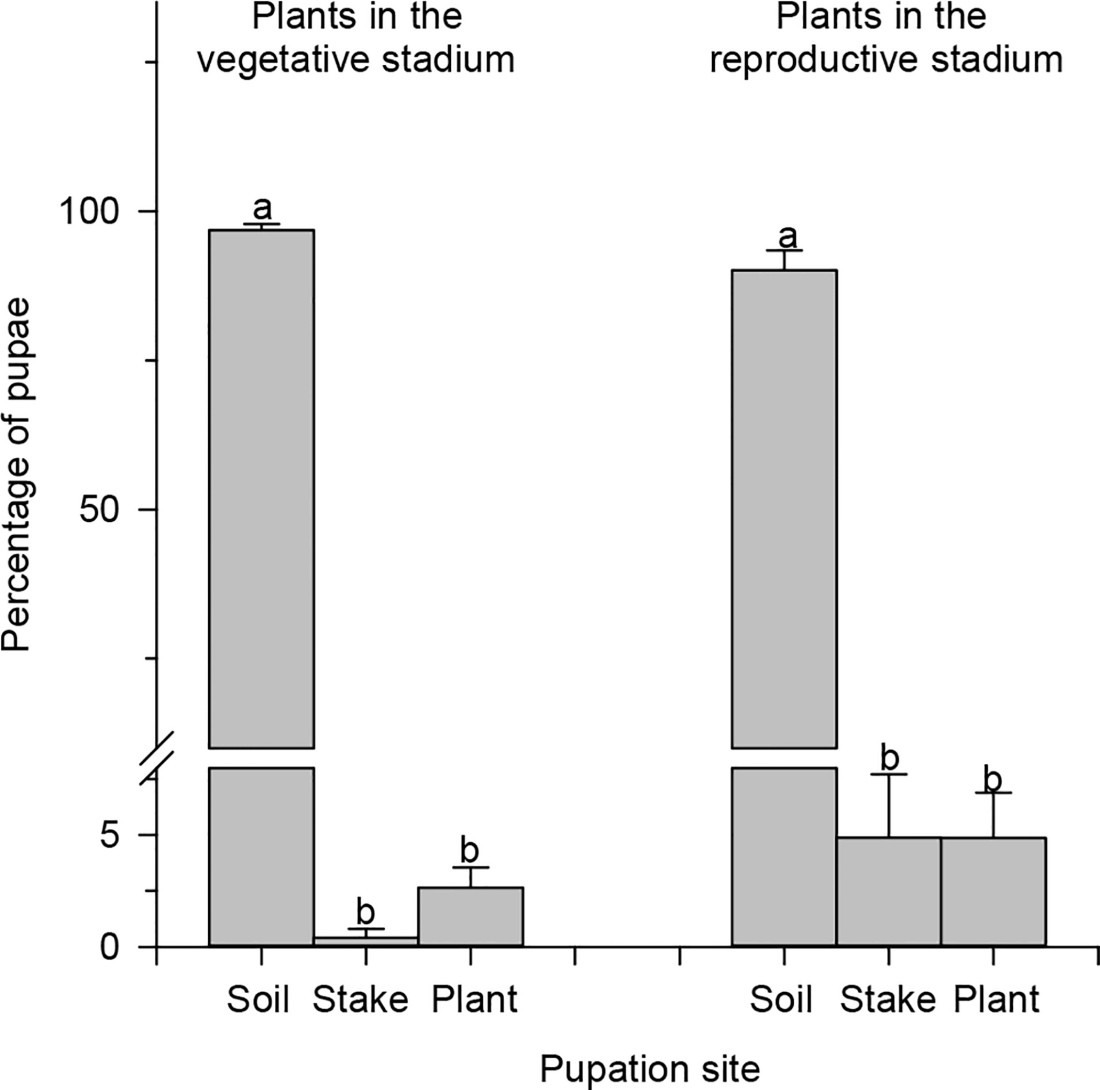
Pupation sites of *Tuta absoluta* on plants in the vegetative and reproductive stages. Histograms followed by the same letter have means that do not differ among themselves by Tukey’s test (p <0.05).

**Fig 7:**
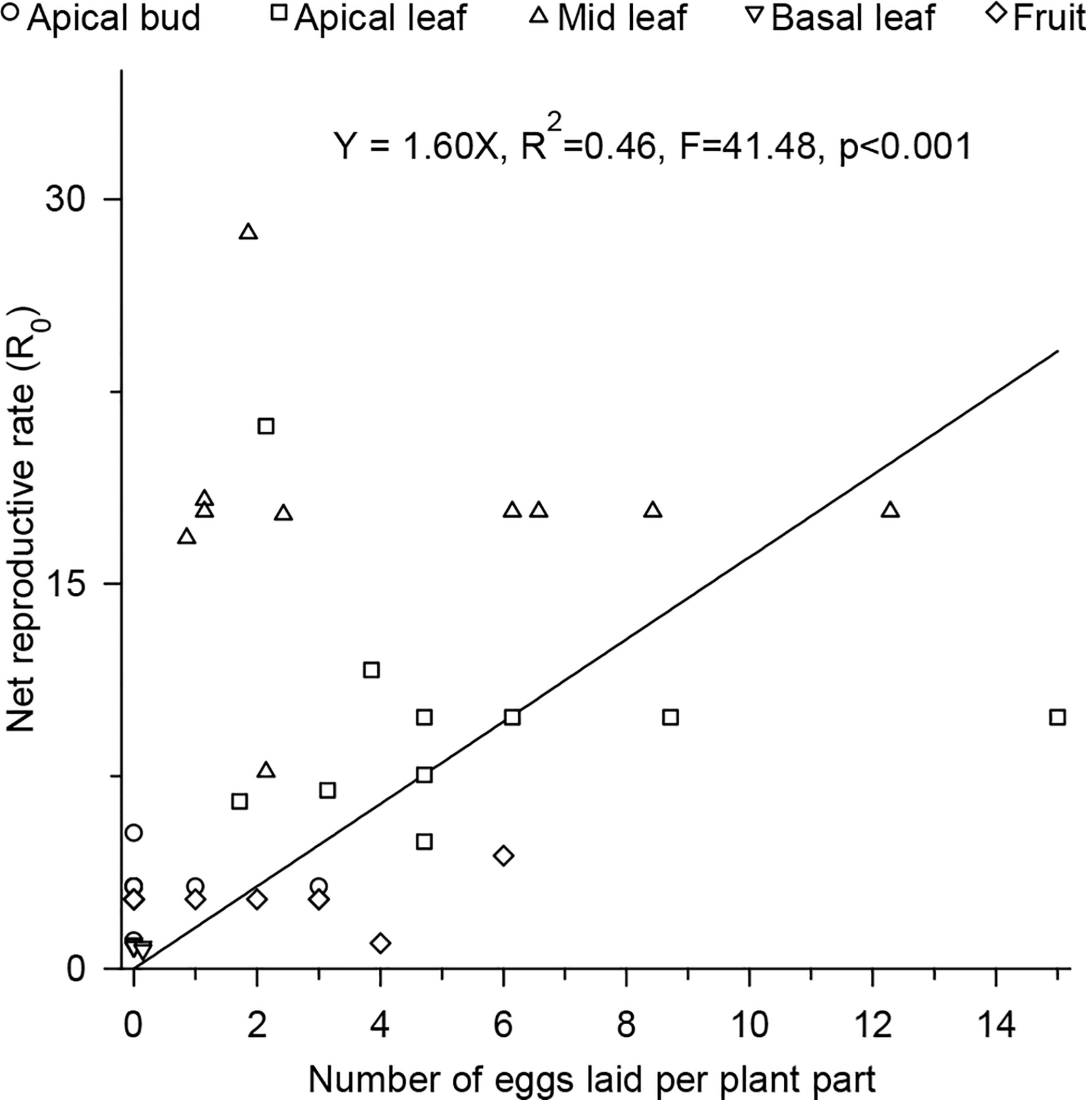
Net reproductive rate in relation to the plant part where the larvae fed.

The main site of pupation of *T*. *absoluta* for plants in the vegetative (96.82% of the pupae) and reproductive (91.10% of the pupae) stages was the soil (Fig 6). Upon performing a regression analysis of the net reproductive rate versus the number of eggs on the plant where the larvae had fed, we found that the cooperative feeding effect resulted in improved performance of this insect species (Fig 7).

## Discussion

Specialist herbivores develop strategies to adapt to their host plant [27], [28]. We observed the behavior of *T*. *absoluta* adult females and larvae in choosing plant parts where their higher performance demonstrated the greatest adaptation of this insect to tomato plants.

The fact that oviposition occurs mainly on the leaf is probably due to the fact that the *T*. *absoluta* larvae feed primarily on the leaf parenchyma, as we observed in this study. Thus, oviposition at sites where the larvae can feed reduces larval mortality in the early instar stages [2], [6], [29]. This effect occurs because the insects in the early instars are less able to move and are more susceptible to unfavorable conditions [4], [30], [31]. This was found to be true in our study as we observed that the *T*. *absoluta* larvae were highly sensitive to starvation and were less able to move during the 1^st^ and 2^nd^ instar stages.

The oviposition rate of *T*. *absoluta* was similar on both leaf surfaces. We expected the majority of the eggs to be oviposit on the abaxial surface where the eggs are better protected from climatic hazards and natural enemies [2], [3], [32], [33]. However, in tomato plants, the behavior of egg laying on both sides of the leaf was also observed for other species [34]. A similar type of oviposition revealed by *T*. *absoluta* on both leaf surfaces may be related to the fact that their larvae are leaf miners. The leaf miners feed on the parenchyma by penetrating into the leaves. In this context, there are species that feed on the palisade parenchyma, while others feed on the spongy parenchyma. However, there are also leaf miners that feed on both of these leaf tissues [31], [35], [36].

The leaf miners whose larvae begin feeding on the palisade parenchyma usually oviposit on the adaxial leaf face because this site is closest to this tissue [31]. For the same reasons, the leaf miners whose larvae begin feeding on the spongy parenchyma preferentially lay their eggs on the abaxial surface [35]. Therefore, by laying eggs on both leaf surfaces, some *T*. *absoluta* larvae can start feeding on the palisade parenchyma, while the other larvae can begin to feed on the spongy parenchyma. This suggests that the joint use of the palisade and spongy parenchyma enables the insect to better exploit this food resource.

The preference of *T*. *absoluta* for oviposition on fully expanded, and not senescent leaves, is possibly because these leaves represent a better food resource for the larvae. These leaves constitute a better food source because they have a higher mass than the unexpanded leaves from the plant apex and higher nutritional quality than the senescent leaves at the basal level of the plant [23], [37]. This can be explained by plant defense theory, one of the assumptions of which is that plants allocate resources to defend parts that are more important to their development and propagation [8], [10]. It seems that fully expanded leaves do not possess many defenses because they are not as important to plant as the apical leafs and fruit [8].

The first instar larvae remain in the oviposition site due to their low capacity to move on the plant and also due to the good nutritional quality of the leaves on which the females had laid their eggs (new leaves). However, over time, these leaves become old and lose their nutritional quality quite fast, which is further induced by the attack of *T*. *absoluta* (either eggs or larvae) [23], [37–39]. This loss results in the third instar larvae (which possess a good capacity for locomotion) moving to the more apical leaves on the plant. This *T*. *absoluta* larval movement to the apical sites as the tomato plants develop shows the adaptation of this insect to its host once the larvae begin to move to the leaves with better nutritional quality.

The high adaptability of *T*. *absoluta* to tomato plants noted in our study is highly beneficial to this species. Insects with better availability of nutrition grow much faster and achieve advanced phases of their development sooner than normal (e.g., pupal stage). Since they develop faster, they stay exposed to natural enemies and fatal biotic and abiotic factors for a shorter time, because of which their chances of mortality due to these factors is highly reduced [40–43]. In the case of the tomato leaf miner *T*. *absoluta*, this fast growth is facilitated by the early selection of good site by the adult female (Fig 1), followed by the movement of larvae to places in the plant canopy with better nutrition (Fig 4). It is highly uncommon for a species to demonstrate both these behaviors (female and larvae choice) at the same time. Normally, when a larva is capable of selecting a site with good food source, the female species is not and vice versa [2], [44]. Nevertheless, in our study, *T*. *absoluta* demonstrated both these behaviors at the same time.

The spatial distribution pattern of the *T*. *absoluta* larvae in the plant canopy assumes great importance in planning integrated pest management programs. One consequence is that sampling of the *T*. *absoluta* larvae should be performed on the leaves of the apical and middle thirds of the crop canopy. Additionally, insecticide spraying should be focused on the leaves of the apical and middle thirds, as these are the sites of major attack by the *T*. *absoluta* larvae on the plants.

The third instar larval movement from the leaves to the fruits possibly occurred because the older *T*. *absoluta* larvae (3^rd^ and 4^th^ instars) were more tolerant to the toxins present in the fruits, similar to observations for other insect species [45], [46]. These instars also probably have stronger mandibles that can facilitate the penetration of larvae into the fruit. The fact that the 3^rd^ and 4^th^ instars became more tolerant to the toxins could be one reason that first instars do not move to apical leaves. Once these leaves are well defended (optimal defense theory), only the 3^rd^ and 4^th^ instars establish well on these leaves. The toxins that are present in higher concentrations in the tomato fruits compared to the leaves include α-tomatine, rutin and dehydrotomatine [14], [15]. Moreover, the fruits have higher nutrient concentrations than the leaves, thus supplying the greatest energy demand of the insects at the end of their larval stages [47].

Attacks by these insects on the fruits results in a greater reduction in crop yields than when they attack leaves [17]. Therefore, we must use control methods that reduce pest attacks before they begin to attack the fruits. For *T*. *absoluta* control, one method of achieving this goal is farmer control of the larvae before the third instar stage, when the insects have not yet migrated to the fruits. Moreover, the *T*. *absoluta* larvae in both the initial instars (first and second) are more susceptible to control methods than they are at the later stages [23], [45], [46]. Additionally, the efficiency of larval control is much higher when the larvae are at the leaf-mining stage rather than when they are at the fruit-boring stage [48].

Because *T*. *absoluta* feeds on both leaves and fruits, it might be assumed that to produce plants resistant to this pest, resistance factors must be incorporated into these two plant organs. However, this approach has its limitations because if the toxins and glandular trichomes that are the main means of resistance of the tomato plants to *T*. *absoluta* were incorporated into the tomato fruits, it would render them unsuitable for human consumption [49]. However, the fact that the larvae that attack the fruits originate from the leaves indicates an alternative method of overcoming this limitation. As shown, these insects mine leaves and walk throughout the plant. In this phase of the developmental cycle of *T*. *absoluta*, trichomes play the most important role in plant resistance against this pest. Trichomes prevent the larvae from walking on the plant, mainly on the stems. All types of trichomes are important in this part of the plant because they serve as a physical barrier to prevent the movement of the larvae. Glandular trichomes are one of the most important defenses against this insect, and they act by releasing chemical components to kill the larvae. Therefore, in the tomato varieties resistant to *T*. *absoluta*, resistance to the pest needs to be incorporated into the leaves and stems in a manner that would control this pest without rendering the fruit unsuitable for human consumption.

Plant breeding programs have long sought to identify the sources of such resistance and then introduce these resistance factors into commercial cultivars. According to our results, we observed that the most vulnerable locations on the tomato plants to *T*. *absoluta* infestation are the leaves of the apical and middle thirds of the canopy, as these sites sustained the maximum performance of this insect pest. Therefore, plant breeding programs should work to develop tomato plants with parts more resistant to *T*. *absoluta*.

*T*. *absoluta* pupation occurred in the soil because this location provided greater protection against pupal desiccation [50], [51] and natural enemies [52].

Failures in the chemical control of *T*. *absoluta* are quite common. Usually, such failures are attributed to the great capacity of the pest to develop resistance to the insecticides [17], [18]. However, larval movement through the plant canopy can also be a primary means that contributes to the failures in the control of this pest. This may occur as a result of the insecticide being sprayed on a plant part where the pest number is low.

Moreover, we discovered that the *T*. *absoluta* females and offspring (larvae) can identify locations on the plants where their performance is greatest. In addition, their offspring (larvae) are distributed spatially in the plant canopy to enable a better development due a better exploitation of the food resource.

## References

[1] Gullan PJ, Cranston PS. The insects: an outline of entomology 4th ed. Oxford: Wiley-Blackwell; 2010.

[2] Thompson JN. Evolutionary ecology of the relationship between oviposition preference and performance of offspring in phytophagous insects. Entomologia Experimentalis et Applicata. 1988;47(1):3–14. 10.1111/j.1570-7458.1988.tb02275.x

[3] Thompson JN, Pellmyr O. Evolution of oviposition behavior and host preference in lepidoptera. Annual Review of Entomology. 1991;36(1):65–89. 10.1146/annurev.en.36.010191.000433

[4] Zalucki MP, Clarke AR, Malcolm SB. Ecology and behavior of first instar larval lepidoptera. Annual Review of Entomology. 2002;47(1):361–93. 10.1146/annurev.ento.47.091201.145220 11729079

[5] Robert CAM, Veyrat N, Glauser G, Marti G, Doyen GR, Villard N, et al A specialist root herbivore exploits defensive metabolites to locate nutritious tissues. Ecology Letters. 2012;15(1):55–64. 10.1111/j.1461-0248.2011.01708.x 22070646

[6] Rajapakse CNK, Walter GH, Moore CJ, Hull CD, Cribb BW. Host recognition by a polyphagous lepidopteran (*Helicoverpa armigera*): primary host plants, host produced volatiles and neurosensory stimulation. Physiological Entomology. 2006;31(3):270–7. 10.1111/j.1365-3032.2006.00517.x

[7] Ali JG, Agrawal AA. Specialist versus generalist insect herbivores and plant defense. Trends in Plant Science. 2012;17(5):293–302. 10.1016/j.tplants.2012.02.006 22425020

[8] Kloth KJ, Thoen MPM, Bouwmeester HJ, Jongsma MA, Dicke M. Association mapping of plant resistance to insects. Trends in Plant Science. 2012;17(5):311–9. 10.1016/j.tplants.2012.01.002 22322003

[9] Knolhoff LM, Heckel DG. Behavioral Assays for Studies of Host Plant Choice and Adaptation in Herbivorous Insects. Annual Review of Entomology. 2014;59(1):263–78. 10.1146/annurev-ento-011613-161945 . 24160429

[10] Johnson MTJ. Evolutionary ecology of plant defences against herbivores. Functional Ecology. 2011;25(2):305–11. 10.1111/j.1365-2435.2011.01838.x

[11] Johnson SN, Birch ANE, Gregory PJ, Murray PJ. The ‘mother knows best’ principle: should soil insects be included in the preference–performance debate? Ecological Entomology. 2006;31(4):395–401. 10.1111/j.1365-2311.2006.00776.x

[12] Antônio AC, Silva DJH, Picanço MC, Santos NT, Fernandes MES. Tomato plant inheritance of antixenotic resistance to tomato leafminer. Pesquisa Agropecuária Brasileira. 2011;46(1):74–80.

[13] Leite GLD, Picanço M, Guedes RNC, Zanuncio JC. Role of plant age in the resistance of *Lycopersicon hirsutum* f. *glabratum* to the tomato leafminer *Tuta absoluta* (Lepidoptera: Gelechiidae). Scientia Horticulturae. 2001;89(2):103–13.

[14] Kennedy GG. Tomato, pests, parasitoids, and predators: tritrophic interactions involving the genus *Lycopersicon* . Annual Review of Entomology. 2003;48:51–72. 10.1146/annurev.ento.48.091801.112733 12194909

[15] Kozukue N, Han J-S, Lee K-R, Friedman M. Dehydrotomatine and α-Tomatine content in tomato fruits and vegetative plant tissues. Journal of Agricultural and Food Chemistry. 2004;52(7):2079–83. 10.1021/jf0306845 15053555

[16] Picanço MC, Bacci L, Crespo ALB, Miranda MMM, Martins JC. Effect of integrated pest management practices on tomato production and conservation of natural enemies. Agricultural and Forest Entomology. 2007;9(4):327–35. 10.1111/j.1461-9563.2007.00346.x

[17] Silva GA, Picanço MC, Bacci L, Crespo ALB, Rosado JF, Guedes RNC. Control failure likelihood and spatial dependence of insecticide resistance in the tomato pinworm, *Tuta absoluta* . Pest Management Science. 2011;67(8):913–20. 10.1002/ps.2131 21394881

[18] Gontijo PC, Picanço MC, Pereira EJG, Martins JC, Chediak M, Guedes RNC. Spatial and temporal variation in the control failure likelihood of the tomato leaf miner, *Tuta absoluta* . Annals of Applied Biology. 2013;162(1):50–9. 10.1111/aab.12000

[19] Desneux N, Wajnberg E, Wyckhuys K, Burgio G, Arpaia S, Narváez-Vasquez C, et al Biological invasion of European tomato crops by *Tuta absoluta*: ecology, geographic expansion and prospects for biological control. Journal of Pest Science. 2010;83(3):197–215. 10.1007/s10340-010-0321-6

[20] Desneux N, Luna M, Guillemaud T, Urbaneja A. The invasive South American tomato pinworm, *Tuta absoluta*, continues to spread in Afro-Eurasia and beyond: the new threat to tomato world production. Journal of Pest Science. 2011;84(4):403–8. 10.1007/s10340-011-0398-6

[21] Silva DJH, Vale FXR. Tomate: tecnologia e produção Viçosa: Editora UFV; 2007. 355 p.

[22] Leite GLD, Picanço MC, Azevedo AA, Zurita Y, Marquini F. Oviposicion y mortalidad de *Tuta absoluta* en *Lycopersicon hirsutum* . Manejo Integrado de Pragas. 1998;22(1):26–34.

[23] Galdino TVS, Picanço MC, Morais EGF, Silva NR, Silva GAR, Lopes MC. Bioassay method for toxicity studies of insecticide formulations to *Tuta absoluta* (Meyrick, 1917). Ciência e Agrotecnologia. 2011;35(5):869–77.

[24] Coelho MCF, França FH. Biologia, quetotaxia da larva e descrição da pupa e adulto da traça-do-tomateiro Pesquisa Agropecuária Brasileira. 1987;22(2):129–35.

[25] Southwood TRE, Henderson PA. Ecological methods, with marticular reference to the study of insect populations Third ed: Oxford: Blackwell Science; 2000.

[26] SAS Institute. SAS/STAT User’s Guide, v.8. SAS Institute, Cary, NC, USA 2001.

[27] Bernays EA. Neural limitations in phytophagous insects: implications for diet breadth and evolution of host affiliation. Annual Review of Entomology. 2001;46:703–27. 10.1146/annurev.ento.46.1.703 11112184

[28] Städler E, Reifenrath K. Glucosinolates on the leaf surface perceived by insect herbivores: review of ambiguous results and new investigations. Phytochemistry Reviews. 2009;8(1):207–25. 10.1007/s11101-008-9108-2

[29] Cunningham JP. Can mechanism help explain insect host choice? Journal of Evolutionary Biology. 2012;25(2):244–51. 10.1111/j.1420-9101.2011.02435.x 22225990

[30] Miranda MMM, Picanço M, Zanuncio JC, Guedes RNC. Ecological life table of *Tuta absoluta* (Meyrick) (Lepidoptera: Gelechiidae). Biocontrol Sci Technol. 1998;8(4):597–606. 10.1080/09583159830117

[31] Pereira EJG, Picanço MC, Bacci L, Della Lucia TMC, Silva ÉM, Fernandes FL. Natural mortality factors of *Leucoptera coffeella* (Lepidoptera: Lyonetiidae) on *Coffea arabica* . Biocontrol Sci Technol. 2007;17(5):441–55. 10.1080/09583150701309337

[32] Renwick JAA, Chew FS. Oviposition behavior in lepidoptera. Annual Review of Entomology. 1994;39(1):377–400. 10.1146/annurev.en.39.010194.002113

[33] Faraji F, Janssen A, Sabelis MW. Oviposition patterns in a predatory mite reduce the risk of egg predation caused by prey. Ecological Entomology. 2002;27(6):660–4. 10.1046/j.1365-2311.2002.00456.x

[34] Alvarado-Rodriguez B, Leigh TF, Lange HW. Oviposition Site Preference by the Tomato Fruitworm (Lepidoptera: Noctuidae) on Tomato, with Notes on Plant Phenologyl. Journal of Economic Entomology. 1982;75(5):895–8. 10.1093/jee/75.5.895

[35] Irvin NA, Hoddle MS. Oviposition preference of *Homalodisca coagulata* for two citrus limon cultivars and influence of host plant on parasitism by *Gonatocerus ashmeadi* and *G*. *triguttatus* (Hymenoptera: Mymaridae). Florida Entomologist. 2004;87(4):504–10. 10.1653/0015-4040(2004)087[0504:opohcf]2.0.co;2

[36] Maluf W, de Fátima Silva V, das Graças Cardoso M, Gomes L, Neto Á, Maciel G, et al Resistance to the South American tomato pinworm *Tuta absoluta* in high acylsugar and/or high zingiberene tomato genotypes. Euphytica. 2010;176(1):113–23. 10.1007/s10681-010-0234-8

[37] Lim PO, Kim HJ, Gil Nam H. Leaf senescence. Annual Review of Plant Biology. 2007;58:115–36. 10.1146/annurev.arplant.57.032905.105316 17177638

[38] Hoy CW, Head GP, Hall FR. Spatial heterogeneity and insect adaptation to toxins. Annual Review of Entomology. 1998;43(1):571–94. 10.1146/annurev.ento.43.1.571 . 15012398

[39] Slansky F, Scriber JM. Food consumption and utilization In: Kerkut GA GL, editor. Comprehensive Insect Physiology, Biochemistry and Pharmacology. 4 Oxford: Pergamon Press; 1985 p. 87–163.

[40] Björkman C, Larsson S, Bommarco R. Oviposition Preferences in Pine Sawflies: A Trade-Off between Larval Growth and Defence against Natural Enemies. Oikos. 1997;79(1):45–52. 10.2307/3546088

[41] Cornelissen T, Stiling P. Does low nutritional quality act as a plant defence? An experimental test of the slow-growth, high-mortality hypothesis. Ecological Entomology. 2006;31(1):32–40. 10.1111/j.0307-6946.2006.00752.x

[42] Scriber JM. Evolution of insect-plant relationships: chemical constraints, coadaptation, and concordance of insect/plant traits. Entomologia Experimentalis et Applicata. 2002;104(1):217–35. 10.1046/j.1570-7458.2002.01009.x

[43] Scriber JM, Feeny P. Growth of Herbivorous Caterpillars in Relation to Feeding Specialization and to the Growth Form of Their Food Plants. Ecology. 1979;60(4):829–50. 10.2307/1936618

[44] Li YX, Liu TX. Oviposition preference, larval performance and adaptation of *Trichoplusia ni* on cabbage and cotton. Insect Science. 2015;22(2):273–82. 10.1111/1744-7917.12104 24431263

[45] Liu TX, Sparks ANJ, Chen W, Liang G, Brister C. Toxicity, persistence, and efficacy of indoxacarb on Cabbage looper (Lepidoptera: Noctuidae) on cabbage. Journal of Economic Entomology. 2002;95(2):360–7. 10.1603/0022-0493-95.2.360 12020014

[46] Wang D, Gong PY, Li MQ, X.H., Wang KY. Sublethal effects of spinosad on survival, growth and reproduction of *Helicoverpa armigera* (Lepidoptera: Noctuidae). Pest Management Science. 2009;65(2):223–7. 10.1002/ps.1672 19097023

[47] Gary C, Bertin N, Frossard J-S, Le Bot J. High mineral contents explain the low construction cost of leaves, stems and fruits of tomato plants. Journal of Experimental Botany. 1998;49(318):49–57. 10.1093/jxb/49.318.49

[48] Miranda MMM, Picanço MC, Zanuncio JC, Bacci L, Silva ÉMd. Impact of integrated pest management on the population of leafminers, fruit borers, and natural enemies in tomato. Ciência Rural. 2005;35(1):204–8.

[49] Friedman M. Analysis of biologically active compounds in potatoes (*Solanum tuberosum*), tomatoes (*Lycopersicon esculentum*), and jimson weed (*Datura stramonium*) seeds. Journal of Chromatography A. 2004;1054(1–2):143–55. 15553139 10.1016/j.chroma.2004.04.049

[50] Ellis JD, Hepburn R, Luckman B, Elzen PJ. Effects of soil type, moisture, and density on pupation success of *Aethina tumida* (Coleoptera: Nitidulidae). Environmental Entomology. 2004;33(4):794–8. 10.1603/0046-225x-33.4.794

[51] Hulthen AD, Clarke AR. The influence of soil type and moisture on pupal survival of *Bactrocera tryoni* (Froggatt) (Diptera: Tephritidae). Australian Journal of Entomology. 2006;45(1):16–9. 10.1111/j.1440-6055.2006.00518.x

[52] Hou B, Xie Q, Zhang R. Depth of pupation and survival of the Oriental fruit fly, *Bactrocera dorsalis* (Diptera: Tephritidae) pupae at selected soil moistures. Applied Entomology and Zoology. 2006;41(3):515–20. 10.1303/aez.2006.515.

